# Integrative Analysis of Multi-Omics Data to Identify Deregulated Molecular Pathways and Druggable Targets in Chronic Lymphocytic Leukemia

**DOI:** 10.3390/jpm14080831

**Published:** 2024-08-06

**Authors:** Dimitra Mavridou, Konstantina Psatha, Michalis Aivaliotis

**Affiliations:** 1 Laboratory of Biological Chemistry, School of Medicine, Faculty of Health Sciences, Aristotle University of Thessaloniki, GR-54124 Thessaloniki, Greece; dimitramd@auth.gr; 2 Functional Proteomics and Systems Biology (FunPATh), Center for Interdisciplinary Research and Innovation (CIRI-AUTH), GR-57001 Thessaloniki, Greece; kpsatha@auth.gr; 3 Basic and Translational Research Unit, Special Unit for Biomedical Research and Education, School of Medicine, Aristotle University of Thessaloniki, GR-54124 Thessaloniki, Greece; 4 Laboratory of Medical Biology—Genetics, School of Medicine, Faculty of Health Sciences, Aristotle University of Thessaloniki, GR-54124 Thessaloniki, Greece

**Keywords:** chronic lymphocytic leukemia, lymphoma, proteomics, transcriptomics, drug repurposing, personalized medicine

## Abstract

Chronic Lymphocytic Leukemia (CLL) is the most common B-cell malignancy in the Western world, characterized by frequent relapses despite temporary remissions. Our study integrated publicly available proteomic, transcriptomic, and patient survival datasets to identify key differences between healthy and CLL samples. We exposed approximately 1000 proteins that differentiate healthy from cancerous cells, with 608 upregulated and 415 downregulated in CLL cases. Notable upregulated proteins include YEATS2 (an epigenetic regulator), PIGR (Polymeric immunoglobulin receptor), and SNRPA (a splicing factor), which may serve as prognostic biomarkers for this disease. Key pathways implicated in CLL progression involve RNA processing, stress resistance, and immune response deficits. Furthermore, we identified three existing drugs—Bosutinib, Vorinostat, and Panobinostat—for potential further investigation in drug repurposing in CLL. We also found limited correlation between transcriptomic and proteomic data, emphasizing the importance of proteomics in understanding gene expression regulation mechanisms. This generally known disparity highlights once again that mRNA levels do not accurately predict protein abundance due to many regulatory factors, such as protein degradation, post-transcriptional modifications, and differing rates of translation. These results demonstrate the value of integrating omics data to uncover deregulated proteins and pathways in cancer and suggest new therapeutic avenues for CLL.

## 1. Introduction

Chronic Lymphocytic Leukemia (CLL) is a blood malignancy marked by the progressive clonal proliferation of B-cell-like cells in the blood, bone marrow, and secondary lymphoid tissues [1,2,3]. Despite advancements in research, significant progress in disease prognosis and the development of therapeutic strategies addressing the diverse genetic profiles and clinical outcomes of CLL remains elusive [4,5,6]. Although patients often initially respond well to treatment and may remain disease-free for years, relapses into active CLL are common, challenging standardized treatment approaches [7]. Therefore, there is an urgent need for a deeper understanding of CLL’s development, progression, staging, and relapse mechanisms, as well as the conception of novel therapies to effectively manage this disease.

While the etiology of CLL remains unknown, certain recurrent chromosomal abnormalities are associated with the disease (e.g., 13q del, 11q del, 17p del, trisomy 12); some may help in disease prognosis (11q del, 17p del), whereas certain gene mutations are considered as drivers of CLL [8,9]. Moreover, CLL cases are classified based on the acquired mutations in immunoglobulin heavy chain variable region gene (IGHV), which categorizes patients in those with “mutated” M-CLL and “unmutated” U-CLL patients [10,11]. M-CLL patients are typically characterized by better clinical outcomes than U-CLL [12]. Moreover, molecular lesions, such as overexpression of zeta-chain associated (ZAP-70) and CD38, as well TP53 dysfunction, are also associated with unfavorable disease outcomes [13,14]. Additionally, another typical feature of this disease is heterogeneity of both symptomatology and pathophysiology, which poses a great challenge for complete disease understanding and treatment [4,7,15]. For instance, while some patients suffer from aggressive disease forms with short survival time, others demonstrate a slow-growing disease with large periods of remission [16,17]. Therefore, accurate detection of the disease, as well as effective treatments are still needed.

The complexity of CLL pathogenesis, mediated by several genetic, epigenetic, transcriptomic, and proteomic alterations, necessitates thorough profiling to determine the disease’s complex molecular basis [18]. The technological advances in the field of sequencing, mass spectrometry, and bioinformatics over the last decades have enabled the multi-omics analysis of samples in CLL. Microarray gene expression studies have helped in characterizing different CLL subtypes through differential deregulated genes and pathways on transcriptome level related to disease development [19,20,21]. Moreover, next-generation sequencing further advanced the resolution of the identified alterations on transcriptome level and helped in grouping genes into large networks (spliceosome, proteasome, and ribosome) that determine disease progression and survival [22]. Nevertheless, transcriptome alterations do not always predict protein changes due, among other factors, to regulatory mechanisms that activate or deactivate the translation of specific transcripts, and therefore proteomic approaches are currently considered as the best approach to elucidate complex disease mechanisms [23]. Proteomics provides valuable insights into cellular conditions, focusing on proteins, the key operators of phenotype and cellular activity [24,25]. Unlike transcriptomics, it uncovers the real-time activities and responses within cells to environmental changes [26]. The human proteome is highly dynamic and diverse, far exceeding the gene count due to alternative splicing and post-translational modifications (PTMs) [27,28,29,30,31,32]. Technological advances enable rapid, both qualitative and quantitative high-throughput proteome analysis, allowing simultaneous sample evaluation and enhancing analytical power [33,34]. Proteomics offers crucial information for disease mechanism elucidation, such as cancer, and supports precision medicine by reflecting a patient’s current state for biomarker discovery and improved treatment decisions [35,36,37,38,39]. Indeed, various studies in the field have analyzed the proteomic landscape of CLL revealing valuable insights and protein alterations related to prognosis and survival, including nucleophosmin [40], Cytochrome c oxidase polypeptide VIb (COXG) [41], LEF-1 [42,43], and hematopoietic lineage cell-specific protein 1 (HS1) [44].

Furthermore, as previously presented by our group, large scale proteomic and transcriptomic data can be utilized to identify FDA approved drugs with therapeutic potential in CLL and MCL (drug repurposing) [45,46]. This strategy of drug identification offers multiple advantages compared to de novo development, including drastically reduced cost, effort, and time [45]. Previous studies have proven numerous compounds efficacious for additional indications, which may be used alone (e.g., acitretin, alitretinoin, and aplidine) or in combination with known CLL drugs, such as clemastine with ibrutinib [47,48].

**Figure 1:**
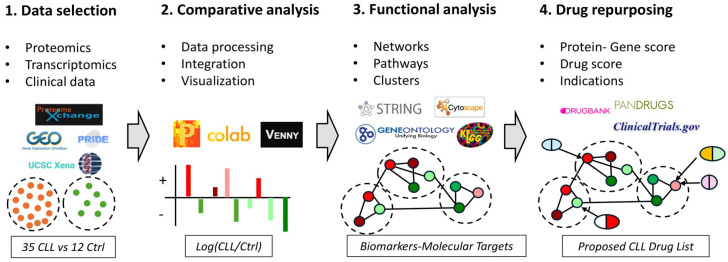
An Integrated Bioinformatics Workflow for Data Analysis and Drug Discovery in CLL. (1) Data Acquisition: The workflow begins with the acquisition of -omics data from various databases including ProteomeXchange, GEO, UCSC Xena, and PRIDE. This step involves collecting-omics data from CLL patients and control groups represented by orange and green dots, respectively. (2) Data Processing and Analysis: The collected data is then processed, integrated and analyzed using tools such as Perseus, Google Colab, and Venny. This step is visualized with bar charts depicting differentially expressed genes or proteins between CLL patients and control groups (red: up-regulated, green: down-regulated). (3). Functional Analysis: Next, functional analysis of the deregulated genes or proteins is performed using databases and tools like STRING, Cytoscape, Gene Ontology, and KEGG. The results are illustrated as interaction networks and pathways between differentially expressed genes or proteins. (4) Drug Discovery: Finally, the workflow integrates drug discovery databases such as DrugBank, PANDRUGS, and ClinicalTrials.gov to identify potential therapeutic targets and existing drugs that could be repurposed. In this step, repurposed drugs are depicted as aligning with specific deregulated pathways and proteins. This comprehensive workflow allows for the systematic integration of multi-omics data, functional analysis, and drug repurposing, facilitating the identification of potential therapeutic targets and treatments in CLL.

The aim of the present study was to integrate proteomic, transcriptomic, and patient survival data to identify deregulated pathways in CLL, as well as propose the expansion of the application of already existing drugs (drug repurposing) in CLL patients. In this context, we utilized three publicly available proteomic data sets that compared the isolated B-cell proteome; in total, 35 patients with 12 healthy controls. We extracted the most significantly deregulated proteins (both increased and decreased) between cancer and healthy cells and identified their impact in patient survival probability (Kaplan–Meyer plots). Next, proteomic data were integrated with transcriptomic data to evaluate their correlation and reveal possible regulation mechanisms in protein expression. We noted transcriptome alterations were associated with corresponding proteomic changes in only a small subset of proteins. We generated protein-protein interaction networks and performed functional enrichment analysis to determine what pathways may promote CLL development. Finally, focusing on the identified pathways as well as the differentially deregulated proteins, we explored how currently approved drugs may be off-label used in CLL patients (Figure 1).

## 2. Materials and Methods

### 2.1. Proteomic Data Selection

Proteomic data of CLL were selected through ProteomeXchange Consortium [49], an open data source that enables internationally coordinated standard data submission and distribution pipelines using the key proteomics repositories, Pride [50], and MassIVE [51]. The search input was either “chronic lymphocytic leukemia” or “CLL”. Only two datasets, PXD002004 and PXD006578, had proteomic data of peripheral B-cell cytoplasm from both CLL patients and healthy controls. To enrich the identified data, the PubMed database was also searched for studies with proteomic data. Except for the studies of Johnston et al. 2018 (PXD002004) [52] and Mayer et al. 2017 (PXD006578) [53], only Thurgood et al. 2019 (proteomic dataset3—PDS3) [54] compared the proteome of peripheral B-cell cytoplasm between CLL patients and healthy controls (Supplemental Table S1), thus this study was also selected. The numbers of CLL patients versus healthy donors in PXD006578, PXD002004, and PDS3 were 9:6, 14:3, and 12:3, respectively. In total, our study reanalyzed proteomic datasets derived from 35 samples patients and 12 healthy individuals.

### 2.2. Transcriptomic Data Selection

Transcriptomic data of CLL were selected using Gene Expression Omnibus (GEO) [55]. GEO is a global public repository that collects and freely disseminates high-throughput gene expression and functional genomics data. The search input was either “chronic lymphocytic leukemia” or “CLL”. Only two datasets, GSE26725 and GSE22529, had transcriptomic data of peripheral B-cell cytoplasm from both CLL patients and healthy controls (Supplemental Table S1). The numbers of CLL patients versus healthy donors in transcriptomic datasets, GSE26725 and GSE22529, were 12:5 and 41:11, respectively. In total, transcriptomic data from 53 samples patients and 16 healthy individuals were integrated with proteomic data in our muti-omics study.

### 2.3. Data Processing and Integration

To compare the proteome of the three studies and integrate them with transcriptomic data, common nomenclature and measurement units were needed. In all studies, the “official name/symbol of gene” was chosen, as well as the “Log2[(measurement of proteins/genes of CLL patients)/(measurement of proteins/genes of healthy donors)]” or “Log2(Fold Change)” or “Log2(FC)” was calculated. If needed, the protein name was converted to gene (official name/symbol) through the BioDBnet [56], an online web resource that provides integrated access to a variety of biological databases and enables the conversions of identifiers from one database to another database identifiers or annotations. Log10(*p* value) was also calculated to statistically evaluate the measurements. Filters of Log2(FC) > 0.3 and Log10(*p* value) > 1.3 were used for the significant deregulated proteins. Processed data from the five different datasets are available in Supplemental Table S2. Data were integrated by Colab, a product from Google Research, that allows anybody to create and execute arbitrary python code directly in the browser, making it ideal for data analysis. The orders of the python code were searched through pandas (2.2.2), a software package that allows high-performance data manipulation in Python. The main work performed in Colab was to merge the excel tables of the different datasets and calculate the means for the same IDs. Interestingly, the dataset GSE22529 had data from two different probe sets, which were therefore re-averaged. Merged data are available in Supplemental Table S3.

### 2.4. Comparative Analysis and Data Visualization

Comparative analysis was conducted using BioVenn [57], an application that compares and visualizes biological lists using area-proportional Venn diagrams. Graphs were made in GraphPad Prism 8 and Excel. Volcano plots were also made in Excel. Scatterplots forms that enable easy visual identification of the proteins’ expression in the three different datasets, highlighting the statistical significance (Log10(*p* value)) versus magnitude of change (Log2(FC)). In order to elucidate the relationship, as well as the difference between the datasets, Principal Component Analysis (PCA) [58] and Hierarchical Clusters—Heatmaps were performed in Perseus-MaxQuant [59], a software that offers a complete framework for statistical analysis of large-scale quantitative proteomics data (http://www.perseus-framework.org, accessed on 25 June 2024). The PCA and Heatmaps providing a visual representation of the differences/similarities between both the three proteomic datasets and transcriptomics are available in Supplemental Figures S1–S3.

### 2.5. Survival Analysis

For the patient survival analysis, we utilized the web-based visual exploration tool UCSC xena [60] that facilitates the study of multi-omic data, such as SNPs and small INDELs, large structural variants, gene-level copy number, segmented copy number, and more. Moreover, it combines data from the main multi-person initiatives that have been completed thus far, including TCGA [61], ICGC [62], and GTEx [63]. For our analysis, we collected the data produced in the ICGC study for each one of the top 10 deregulated proteins. We used “the copy number variation” option that compared patients with high expression of the protein of interest vs. those with low expression (using a custom cutoff value for each protein). Figures and statistical values were created using the built-in tool of the Xena platform More specifically, the Xena Browser employs the log-rank test to compare the Kaplan–Meier curves. The Xena Browser reports the test statistics (*χ*^2^) and *p*-value (*χ*^2^ distribution).

### 2.6. Functional/Pathway Enrichment Analysis

Networks and pathways of the deregulated proteins/genes were generated by String DB [64] and Cytoscape [65]. String DB predicts protein-protein interactions, displays the score of data support, and may cluster data points based on similarity to reveal the underlying structure of data. Clustering was made based on k-means with six clusters in the proteins of increased and decreased levels, and with three clusters in the top five up- and down-regulated proteins, to unravel the most important deregulated pathways. The co-expression scores were also provided by String DB. Specifically, the interactors of the five up- and down-regulated proteins provided by STRING were also searched in our merged multiomics data to find possible deregulation, either in proteomics or transcriptomics level. Cytoscape is also an open-source bioinformatics software platform that allows the visualization of molecular interaction networks (https://cytoscape.org/, accessed on 25 June 2024). GOlorize [66] tool was used to portray the Gene Ontology (GO) categories that were statistically overrepresented in the up- and down-regulated sets of proteins, respectively.

### 2.7. Drug Repurposing

The most significantly upregulated proteins were aligned to repurposed drugs by Pandrugs [67], a bioinformatics tool that prioritizes anticancer drug therapies based on individual genomic data. The options used were drugs that are used both in cancer and other pathologies, and are either FDA approved or in clinical trials, and interact directly with the desired target or the deregulated pathway. The drug list found is in the Supplemental Table S4. Drug candidates with gene score > 0.6 and drug score > 0.7 were selected as best candidates. The evaluation of these drugs was further assessed based on key pharmacological criteria, including potential indications for CLL, avoidance of chemotherapeutic agents, FDA approval in similar cancer types, primary mechanism of action, and the number of targets identified.

## 3. Results

### 3.1. Proteomic Data Integration and Processing Reveal Major Differences between Healthy and CLL Cells

For our proteomics analysis, we incorporated data from three independent studies that used B-cells originating from CLL patients and healthy donors [52,53,54]. The data were generated with both label (PXD002004) and label-free techniques (PXD006578, PDS3) and by different MS/MS instruments. The age of the study population ranged between 60 and 73 years old, which corresponds to the average age of diagnosis. In total, we included data from 35 patients and 12 healthy individuals, hence our study represents the largest comparative analysis (between healthy and cancer samples) in the field to date.

**Figure 2:**
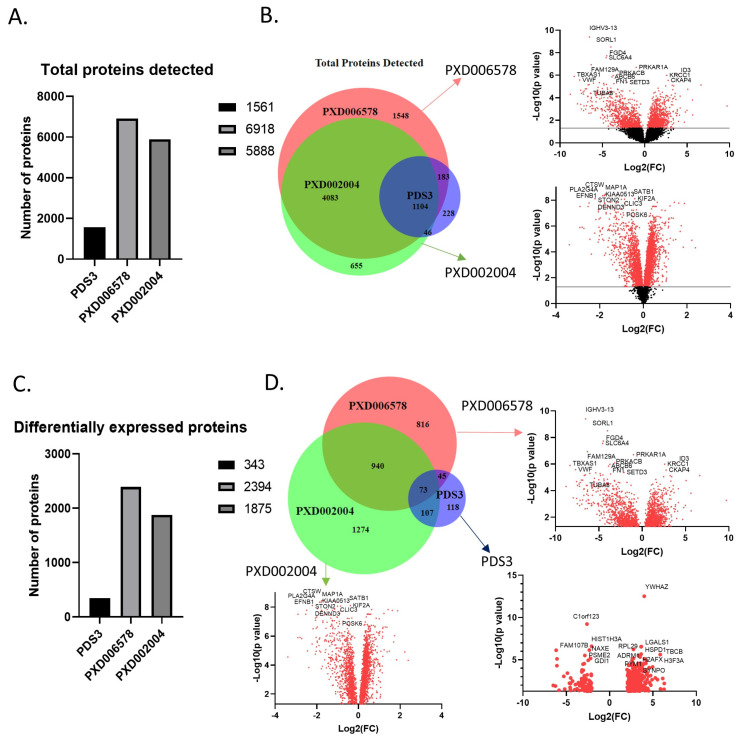
Proteins detected in the three selected proteomics datasets. (**A**) Total proteins detected in each dataset. The small number of proteins identified in the PDS3 dataset is due to the different approach. (**B**) Venn diagram and volcano plots of the total proteins detected. Venn diagram shows the common proteins identified in the three datasets, covering almost 70% of the proteins. Volcano plots show the deregulation of the proteins in relation with the probability. PDS3 has no volcano plot cause the whole data were not publicly available. (**C**) Differentially expressed proteins in each dataset. Filters used are *p*-value < 0.05 and log2(FC) > 0.3 (0.1). (**D**) Venn diagram and volcano plots of the differentially expressed proteins. 1165 proteins were detected in at least two datasets. The red dots in the volcano plot indicate significant protein detection, whereas black dots not significant.

**Figure 3:**
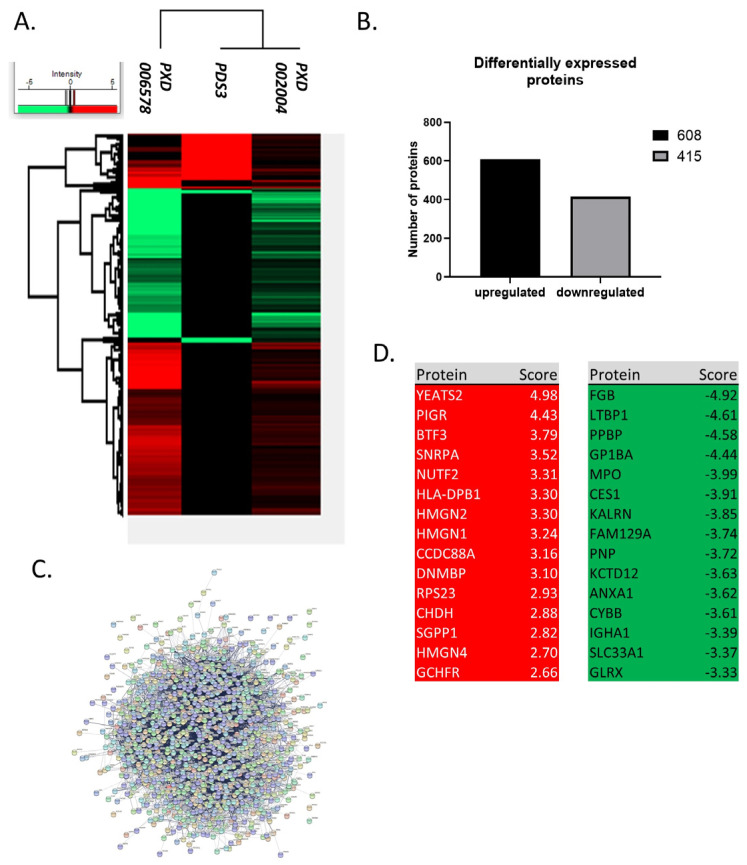
Differentially expressed proteins in CLL. (**A**) Heatmap of differentially expressed proteins in CLL. There are 1023 differentially expressed proteins between healthy and CLL samples detected in at least two datasets and modified in the same direction (up or down-regulation) between datasets. (**B**) Number of deregulated proteins in CLL. 608 proteins are up-regulated and 415 are downregulated. (**C**) Protein-protein interactions of the deregulated proteins. There are strong interactions between proteins that are deregulated in CLL. (**D**) Top 15 up- and down-regulated proteins. The deregulated proteins include several known candidates implicated with both the initiation and the progression of CLL, such as FAM50A, IKZF3, KRAS, MAP2K1, SAMHD1 and SF3B1. Top 15 upregulated proteins have a 6–32 fold increase, while down regulated proteins have a 10–30 fold decrease (score: Log2(FC)).

Two of the three studies included in our analysis reported the identification of a similar number of proteins (6920 and 5888), whereas the third detected 1575. This diversity may be due to the different identification method utilized by the Thurgood et al. group (PDS3), who employed a SWATH approach (Sequential Window Acquisition of all Theoretical Spectra) [68,69], as well as due to differences in the identification criteria used by the groups. Moreover, discrepancy was observed in the analysis of differentially expressed proteins, as ~2000 proteins were referenced in two studies, while only 343 were reported in the third (*p*-value < 0.05, log2(FC) > 0.3 (0.1)). Given the considerable variance in the number of reported proteins across datasets, we decided to include proteins changed in the same manner (up or down-regulation) in at least two of the three studies. The analysis identified 1023 differentially expressed proteins (Figure 2) between healthy and CLL samples, with 608 upregulated and 415 downregulated in CLL (Figure 3). In terms of protein-protein interaction, all proteins were found to be highly connected (Figure 3C). Moreover, the generated list of proteins included several known biomarker candidates implicated with both the initiation and the progression of CLL, such as FAM50A [70], IKZF3 [71], KRAS [72], MAP2K1 [73], SAMHD1 [74], and SF3B1 [75]. We also noted that the degree of deregulation is higher in PXD006578, albeit the rest of the datasets exhibit a similar pattern (Figure 3A). Overall, our results support the notion that the concerted action of 1023 proteins can distinguish cancer from healthy cells and promote tumorigenesis.

### 3.2. Identification of Proteins with Prognostic Nature in CLL Development

**Figure 4:**
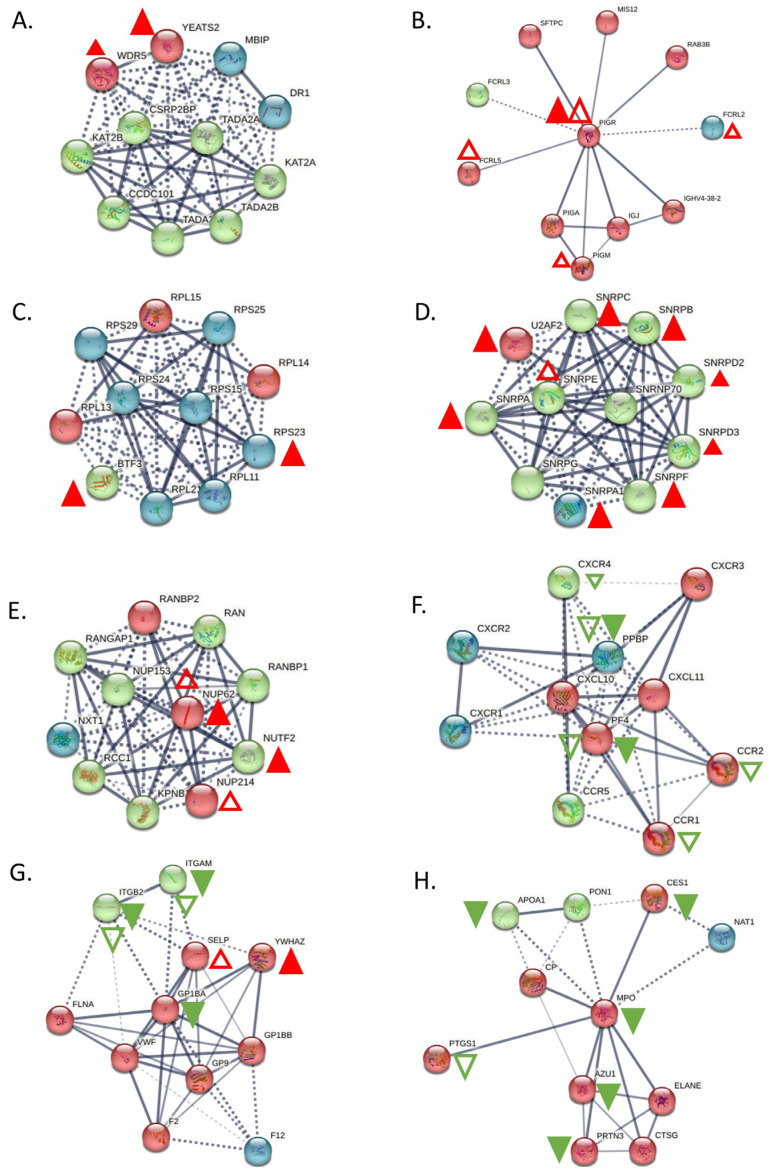
Main interactors of the top deregulated proteins and how they are affected in CLL. (**A**) Interactors of YEATS2. YEATS2 seems to be co-expressed with its already known interactor WDR5, only at the proteomic level. (**B**) Interactors of PIGR. PIGR is upregulated both in proteomic and transcriptomic level, whereas its interactors, FCRL5, FCRL2, and PIGM, were upregulated only at the transcriptomic level. (**C**) Interactors of BTF3. BTF3 seems to have co-expression with RPS23 at the proteomic level. (**D**) Interactors of SNRPA. SNRPA has co-expression with the most of its interactors (SNRPA1, SNRPB, SNRPC, SNRD2, SNRD3, SNRPF and U2AF2) at proteomic level and only one interactor, SNRPE, found to be upregulated at transcriptomic level. (**E**) Interactors of NUTF2. NUP62 was only found up-regulated both at proteomic and transcriptomic level and NUP214 was up-regulated at transcriptomic level. (**F**) Interactors of PPBP. PPBP was also found down-regulated at transcriptomics level, as many of its interactors (CCR1, CCR2, CXCR4 and PF4), whereas only one interactor, PF4 was down-regulated at proteomics level. (**G**) Interactors of GP1BA. Two of its interactors, ITGAM and ITGB, were also found downregulated both at proteomic and transcriptomic level, while two, YWHAZ and SELP had opposite behavior. (**H**) Interactors of MPO. MPO seems to co-expressed with AZU1, PRTN3, APOA1 and CES1, whereas PTGS1 was also found deregulated at transcriptomic level. Red triangles depict over-expression, Green depicts under-expression, Colored filled triangles depict deregulation at proteomic level, Plain triangles depict deregulation at transcriptomic level. The thickness of the lines correlates with the confidence (the strength of the data support) in the network connections between proteins (solid lines: connections with high confidence; dotted lines: connections with lower confidence).

**Figure 5:**
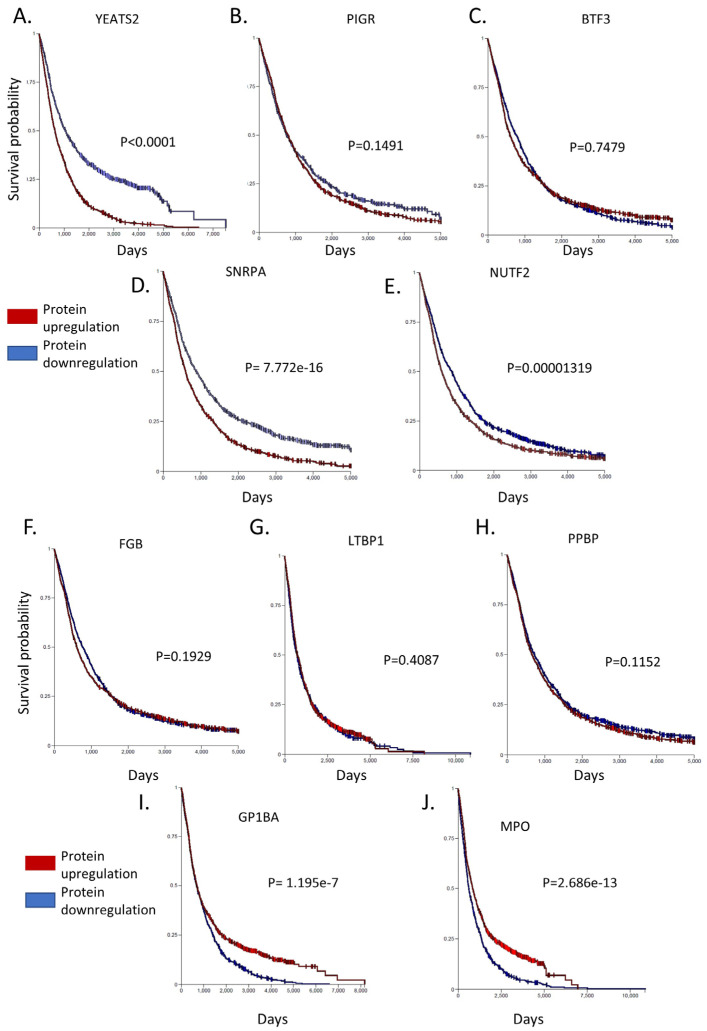
Survival-Kaplan–Meier curves of the top 10 deregulated proteins in CLL. Among the top 5 up-regulated proteins, (**A**) YEATS2, (**B**) PIGR, (**D**) SNRPA and (**E**) NUTF2 have a lower survival probability in CLL patients, apart from (**C**) BTF3 that seems to affect survival only during the initial days of disease development. Among the top 5 down-regulated proteins, (**F**) FGB, (**G**) LTBP1 and (**H**) PPBP seem to not affect the survival curves, while (**I**) GP1BA and (**J**) MPO, when they are downregulated, heavily affected patient survival. Xena Browser compares the different Kaplan–Meier curves using the log-rank test. The Browser reports the test statistics (*χ*^2^) and *p*-value (*χ*^2^ distribution).

Some of the listed proteins may play a more significant role in CLL development over others. To determine such candidates, we selected the top 20 proteins that show the highest deregulation score in CLL cells. By this analysis, we found that YEATS domain containing protein 2 (YEATS2), a chromatin reader, is the most significantly upregulated protein in patients’ cells, with a log2 fold-change equal to 4977. YEATS2 has previously been found to promote tumorigenesis in lung cancer by colocalizing with H3K27 acetylation, facilitating the transcription of essential genes for cell multiplication [76]. However, as the role of this protein in CLL remains unclear, we sought to identify the impact of its upregulation in CLL patients. For this purpose, we calculated how the protein affects the probability that a patient will survive up to a certain time point (survival-Kaplan–Meier curves). Interestingly, we found that patients with high expression of YEATS2 had a markedly lower survival probability compared to patients with low expression (*p* < 0.0001). This profound effect is evident already from the initialization of tumor development and lasts until the final stages. Hence, YEATS2 is likely an important factor in CLL development, as with lung cancer, and further investigation is required to elucidate its role. Furthermore, WDR5, which is one of the proteins, interactors of YEATS2 (they both participate in the Ada2/Gcn5/Ada3 transcription activator complex) and its primary co-expression factor (score 0.089), was found to be around 150% upregulated (Figure 4A). In a similar fashion, Polymeric Immunoglobulin Receptor (PIGR) was also found significantly upregulated in CLL cells, as it is in many other types of cancer [77]. In accordance with YEATS2, PIGR upregulation seems also capable of reducing survival probability in CLL patients’ samples, albeit to a lesser extent than YEATS2 and without statistical significance. Additional proteins with significant upregulated scores (BTF3 [78], SNRPA [79], and NUTF2 [80]), which promote cancer in other types of malignances, also lead to lower survival probability in CLL patients (Figure 5), apart from BTF3 that seems to affect survival only during the initial days of disease development. Interestingly, proteins, RSP23, NUP62, and SNRPA1, SNRPB, SNRPC, SNRD2, SNRD3, SNRPE, SNRPF, and U2AF2, which are known to be associated with BTF3, NUTF2, and SNRPA, respectively, were also found upregulated (Figure 4).

On the contrary, various proteins showed significant levels of downregulation, including FGB, LTBP1, PPBP, GP1BA, and MPO. Of these proteins an interesting candidate is Glycoprotein Ib Platelet Subunit Alpha (GP1BA), a surface membrane glycoprotein with a major function in platelet adhesion during a vascular injury [81]. GP1BA is not only significantly downregulated in our proteomic dataset but also patients with this alteration have a significantly lower survival probability compared to patients with higher GP1BA expression (*p* = 1.195 × 10^−7^). Correspondingly, Myeloperoxidase (MPO) protein is another protein that demonstrated reduced expression in CLL cells and heavily affected patient survival from the initial stages of disease development (*p* = 2.686 × 10^−13^). MPO is a heme protein that serves as a major component of neutrophils, and high MPO expression was associated with favorable outcomes in patients with acute myeloid leukemia (AML) [82]. Interestingly, proteins that are known have co-expression with GP1BA (ITGB2 and ITGAM) and MPO (AZU1 and PRTN3) were also found to be downregulated (Figure 4). Finally, the rest of the proteins investigated (FGB, LTBP1, PPBP) had only a subtle effect in patient survival (with no statistical significance between groups with high and low expression), hence implying that they may act indirectly in CLL development. Nevertheless, the coordinated analysis of proteomics and patient survival data revealed dependence between these two variables and allowed us to identify prominent factors that may act as drivers of disease progression.

### 3.3. Integration of Proteomic Data with Transcriptomics Shows Limited Correlation

The cumulative analysis of many types of omics data has the capacity to significantly improve the molecular characterization of a given cellular state, unveiling the underlying complexity needed for disease subtyping. Thus, we attempted to compare our proteomic data with high-quality transcriptome data to obtain a more thorough picture of the molecular imbalances existing in patient cells. For this purpose, we selected two datasets, GSE26725 and GSE22529, that contain transcriptome data generated through microarrays using 12 and 41 patient samples, respectively. First, we performed data pre-processing by averaging the values of the different probesets that measure the same transcript. Thus, GSE26725 had 30804 and GSE22529 referenced 25,959 identifiers, both of which were substantially larger than the number of proteins identified in proteomic experiments due to the targeting of non-coding areas of the genome. Approximately 81% of the identified loci were found common in both datasets and another 15% was unique in the larger GSE26725 dataset. After integrating the identifiers found in the proteomic datasets, only 3.21% (994) of the transcripts had a protein counterpart, while only 0.02% of the differentially expressed proteins were not detected in the transcriptomic data.

**Figure 6:**
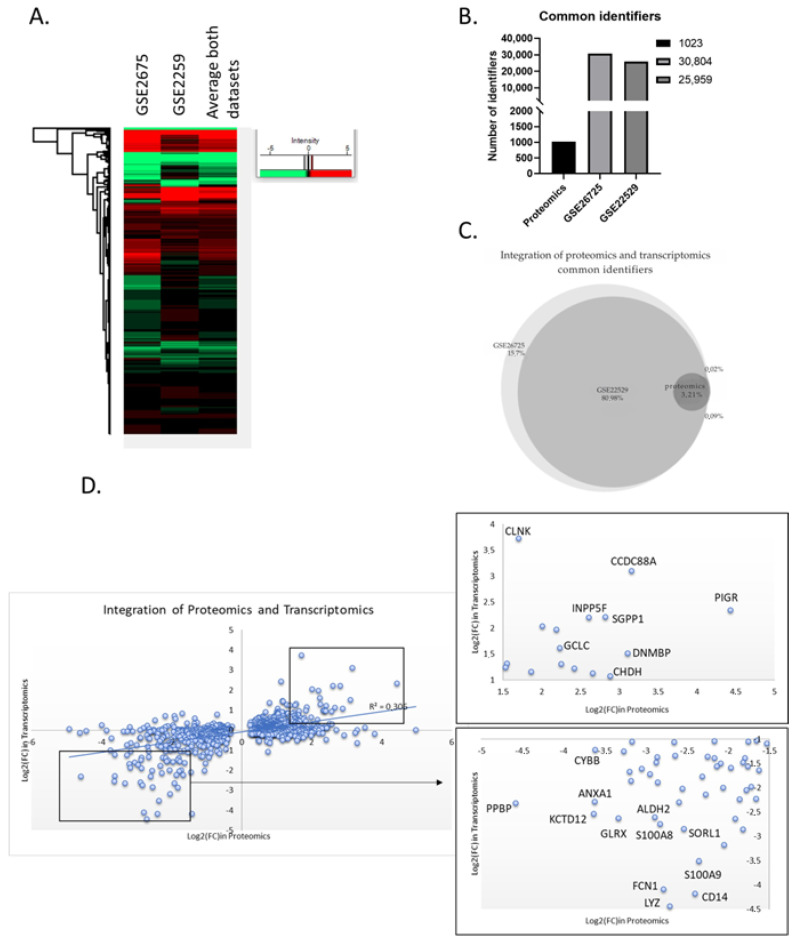
Integration of proteomics and transcriptomics data. (**A**) Heatmap of the two selected transcriptomic datasets. The two datasets display a similar transcriptome profile. (**B**) Number of identifiers in each dataset used for the data integration. “Proteomics” represents the list of the 1023 common differential expressed proteins. (**C**) Venn diagram of the two transcriptomics datasets and the proteomics list. Essentially, the list of differentially expressed proteins “fished” their genes from the transcriptomics datasets. (**D**) Scatter plot of the common identifiers at both proteomics and transcriptomics level. Zoom in the most up- or down-regulated proteins that have common regulation at both proteomic and transcriptomic level.

As previously observed, transcript levels may not be a reliable indicator of protein levels due to a variety of cellular mechanisms that buffer protein biosynthesis (including alternative splicing, transcript stability, translational efficiency, and others). Hence, to examine how transcript levels connect with their protein counterparts, we integrated all datasets with their quantitation values into a single graph. We observed only a modest agreement between proteomic and transcriptomic data, since only a minority of the proteins show the exact same regulation in both mRNA and protein level, while the majority show a higher degree of deregulation in the one type of data over the other (r^2^ = 0.306). Nevertheless, a group of significantly upregulated proteins found in the proteomic dataset were, likewise, strongly upregulated in the transcriptomic dataset (CCDC88A, INPP5F, PIGR, DNMBP, SGPP1, CHDH, GCLC, and CLNK). Similarly, a group of downregulated proteins showed strong concordance with the transcriptomic data (LYZ, FCN1, CD14, S100A9, S100A8, SORL1, ALDH2, GLRX, ANXA1, KCTD12, CYBB, and PPBP). Conversely, there was no consistency between the transcriptomic and proteomic data for YEATS2, which had the highest deregulation score in our proteomic dataset (Figure 6), perhaps indicating a transcript independent regulation of YEATS2 in CLL. On the other hand, PIGR, the second highest deregulated protein, was found increased at the transcriptional level, as were the only two genes, FCRL5 and FCRL2, known to be co-expressed with PIGR (scores 0.070 and 0.076, respectively, Figure 6B). Conclusively, this analysis showed the added value of combining both proteomic and transcriptomic data in elucidating complex disease mechanisms.

### 3.4. Protein-Protein Interaction Network Analysis Reveals Affected Cellular Processes in Tumor Cells

**Figure 7:**
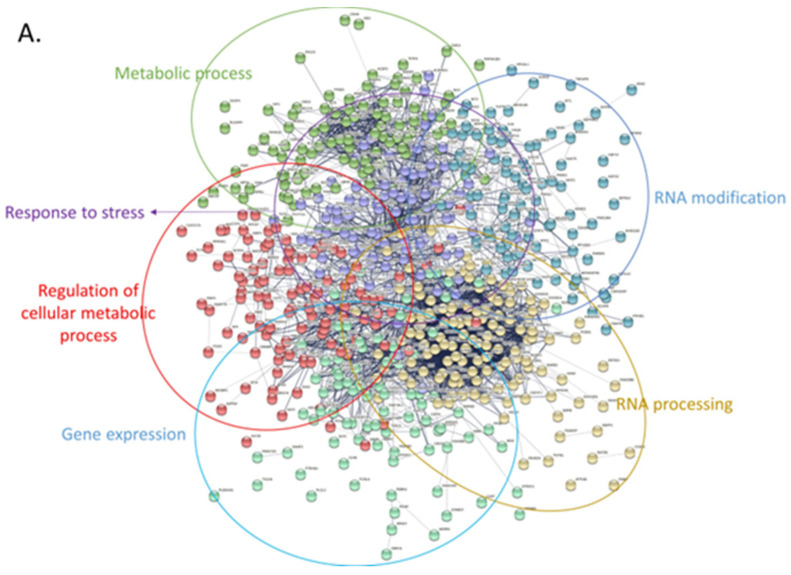

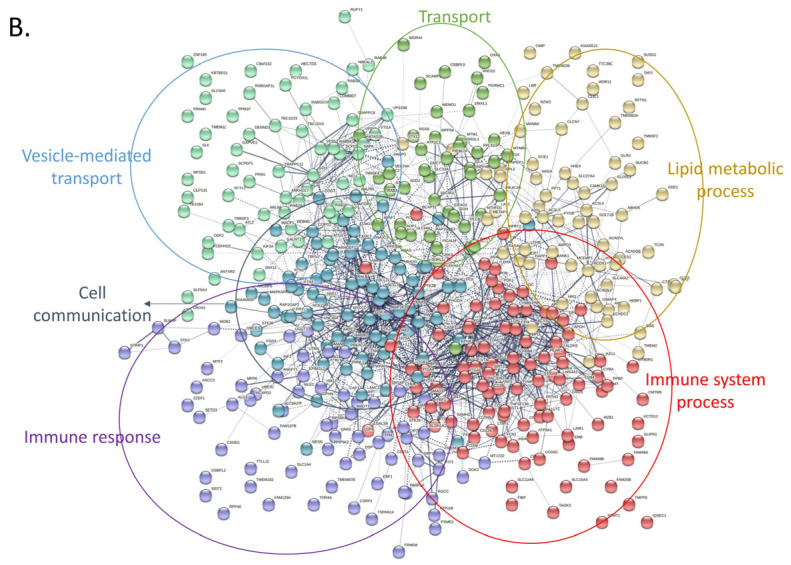
Protein-protein interaction networks of upregulated and downregulated proteins using stringDB. For both networks the following parameters were used: interactions characterized by confidence (instead of only evidence) and clustering of all proteins based on k-means. (**A**) Only upregulated proteins were used to create this network. Proteins were grouped into 6 main clusters and overrepresentation analysis was performed using the built-in stringDB tool. Each cluster was manually labeled with the function (or pathway) mediated by the proteins belonging in that cluster. (**B**) Same analysis as with (**A**), using the downregulated set of proteins.

Next, we attempted to obtain insights into the functionality of the differentially expressed proteins by clustering them based on protein-protein interactions using two distinct bioinformatic tools (String DB and Cytoscape). First, we used String DB, a web-based tool that incorporates known and predicted protein-protein interactions from over 20 million proteins out of 5090 organisms [83]. We selected interactions characterized by confidence (rather than just evidence) and clustered all proteins based on k-means. In total, 608 upregulated proteins were grouped into 6 densely populated clusters with functions related to stress response, RNA modification, metabolism, and gene expression (string DB, k-means clustering, Figure 7A). The RNA modification cluster contained 41 proteins participating in the spliceosome pathway with various roles and functionality across the process of RNA degradation (Figure 7A). Another group consisted of eight proteins related to the synthesis of amino acids, while a third one appeared to confer cellular resistance to stress. Notably, this latter group included prominent members of the heat-shock and the antioxidant response, such as HSPA4, HSPA8, HSPA9, and SOD1. On the contrary, the 415 downregulated proteins were likewise grouped into 6 large clusters with the primary functional enrichments being lipid metabolism, immune system response, as well as cell communication (Figure 7B). Perhaps the most interesting subcluster was the immune system response that seems to confer resistance to Leukocyte- mediated clearance by downregulating the integrin members ITGAM, ITGB1, ITGB2, and ITGAL. Moreover, cellular transport was also compromised in tumor cells through downregulation of 38 proteins related to vesicle-mediated transport, as well as important proteins involved in vesicle docking involved in exocytosis (such as VPS33B, RAB3D, VAMP3, and SCFD1). Finally, downregulated proteins seem to rewire the normal lipid metabolic program in CLL cells, likely promoting energy production through free fatty acids in a similar mechanism to that seen in adipocytes (as previously shown in [84]).

**Figure 8:**
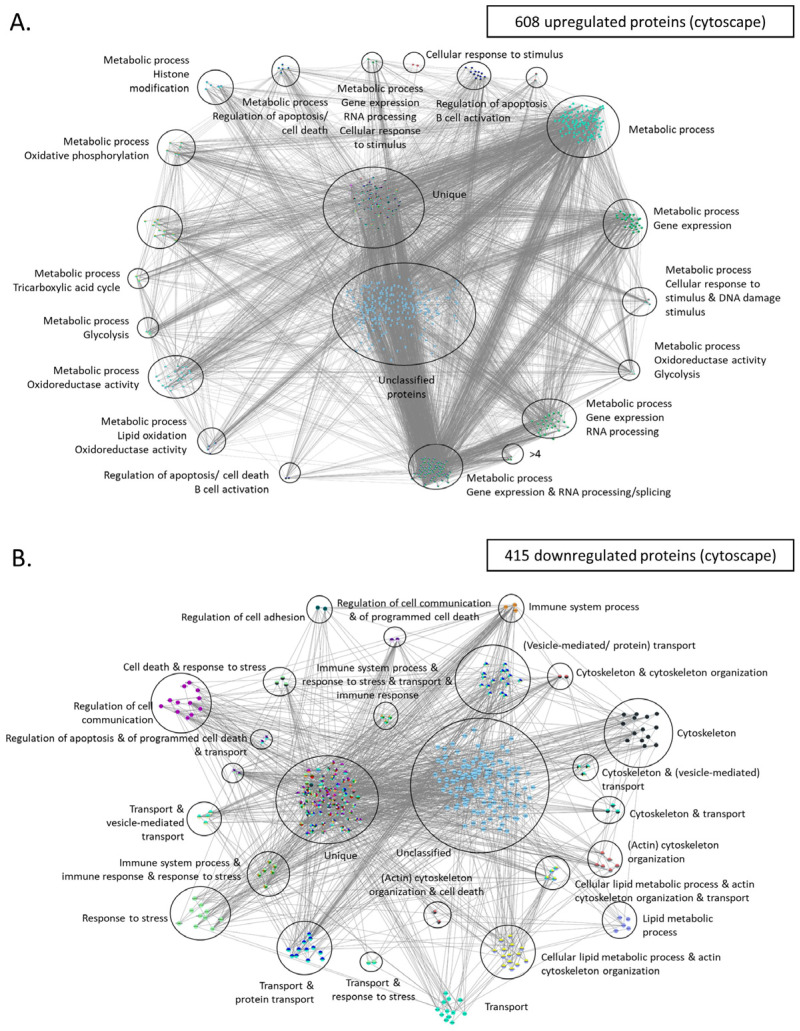
Protein-protein interaction networks of upregulated and downregulated proteins using cytoscape. (**A**) Upregulated proteins were used to create this network. GOlorize tool was used to visualize the Gene Ontology (GO) categories which are statistically overrepresented in the upregulated set of proteins. Each cluster was manually labeled with the function (or pathway) mediated by the proteins belonging in that cluster. (**B**) Same analysis as with (**A**), using the downregulated set of proteins. Unique refers to proteins that are categorized into multipleGO categories. These proteins are considered unique because there is no other protein classified into the exact same combination of GO categories. This distinct classification underscores their unique functional annotations within the dataset.

Moreover, we utilized Cytoscape, which is an open-source software platform that facilitates visualization of protein networks and their integration with annotations, gene expression, and other types of data [65]. These are similar to string DB proteins clustered in various groups using the independent tool of cytoscape. For instance, upregulated proteins were found again to relate with metabolic processes, cellular response to stimulus and DNA damage, and regulation of apoptosis/cell death (upregulated proteins, Figure 8A). The latter group is composed out of 42 proteins (such as GLO1, XIAP, PHB, FOXO1, HSPD1, and TRAF1) and seem to drive evasion of cell death, a trait which represents a main hallmark of cancer. Moreover, another large cluster, composed of 350 proteins, was related to cell metabolism, including processes such as tricarboxylic acid cycle, oxidoreductase activity and glycolysis. The same analysis for downregulated proteins showed that these factors coordinate regulation of cell adhesion, immune system processes, and cytoskeleton organization (Figure 8B). The latter cluster is interesting as it probably confers plasticity and resistance of the cancer cell in both mobility and metastasis. Finally, other major functions mediated through the group of downregulated proteins include stress response, vesicle mediated transport, and regulation of cell communication.

### 3.5. Drug Repurposing Shows Potential New Treatments against CLL

**Figure 9:**
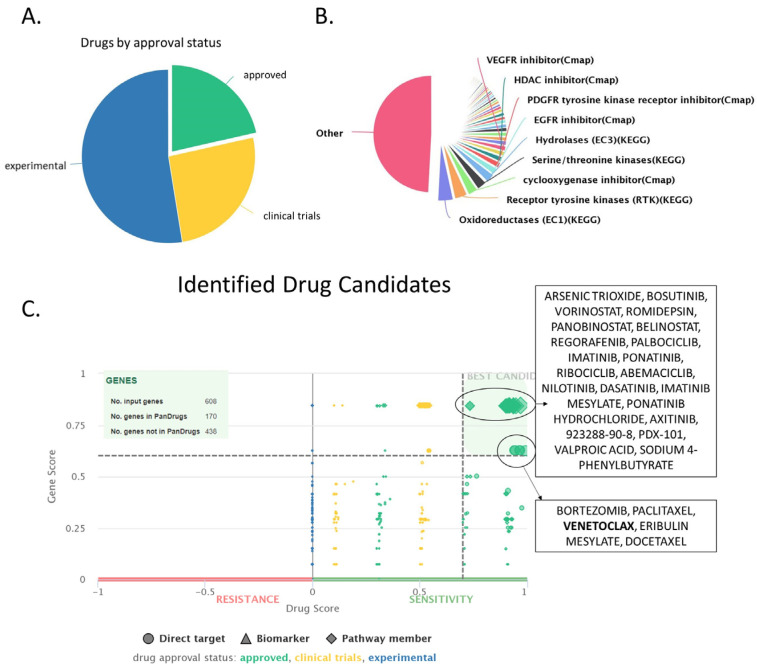
Drug identification using the Pandrugs software (version: 2024.06), based on the upregulated set of proteins (608 proteins). (**A**) Pie chart depicting an overview of the approval status (approved, experimental or in clinical trials) of the identified compounds. (**B**) Overview of the main drug families that the identified compounds belong in. (**C**) Drug score chart depicting all identified compounds separated based on their dscore (considers factors such as approval status, number of associated genes and numbers of sources, *x*-axis) and gscore (considers the importance of the targeted genes for the cancer cell, *y*-axis). Drugs with high g- and d-score are characterized as best candidates, and they are depicted in upper right corner of the graph.

The group of upregulated proteins confers the main traits of cancer cells and differentiates them from healthy one. Thus, we used this group of proteins to identify candidate compounds for treatment in CLL patients. For this purpose, we have used Pandrugs, a bioinformatic tool that incorporates thousands of drug-target associations obtained from 4703 genes and 9073 unique compounds. In our analysis, we mainly focused on approved compounds and excluded experimental or clinical trial candidates so that the identified drugs may be easily implemented in the clinical practice surpassing the need for new clinical trials. Moreover, we selected only the best candidates, characterized by increased sensitivity against a significantly deregulated protein. We identified a total of 782 potential drugs, with the majority of them (601 drugs) targeting a member of a deregulated pathway, and a smaller group of 181 drugs directly targeting a specific protein (Supplemental Table S4, Figure 9). Among them, 411 drugs (52.6%) are currently in an experimental stage, 230 drugs (26%) are in clinical trials, and 168 (21.5%) are approved drugs. Additionally, our drug candidates also target significant enzyme families, such as oxidoreductases, receptor tyrosine kinases, cyclooxygenase inhibitors, serine/threonine kinases, and others. Notably, among the identified drugs, we found a known compound that is already used against CLL, the bcl-2 inhibitor venetoclax, a finding that validates our data analysis.

PanDrugs ranks identified compounds based on two criteria: dscore, which considers factors such as approval status, number of associated genes, and numbers of sources, and gscore, which takes into account the importance of the targeted genes for the cancer cell. Based on both these metrics, the identified drugs were categorized into two groups. The first group contained compounds characterized by high gscore and relatively high sensitivity (but lower than group 2) against the target protein. Group 2 drugs exhibited even higher sensitivity against their target proteins but demonstrated a lower gscore compared to group 1. Another important attribute is that drugs from group 2 target directly a specific protein, while drugs from group 1 may have a direct target but also may target a protein associated with the protein of interest (that belongs in the same pathway) (Table 1, Figure 9).

Thus, the best hits of group 1 were arsenic trioxide, bosutinib, vorinostat, romidepsin, panobinostat, and regorafenib. Interestingly, all these drugs are already approved for the fight against various forms of blood cancer, such as promyelocytic leukemia [107], CML [108], Cutaneous T-cell lymphoma [109], and multiple myeloma [110] (Table 1) [107]. Arsenic trioxide affected eight upregulated proteins in our dataset (CARD11, CDC37, CDKN1B, PRKCB, PTPN2, RUVBL1, SMAD2, and TRAF3). Moreover, bosutinib, a tyrosine kinase inhibitor, is perhaps the best candidate identified, as it is already in use against chronic myeloid leukemia [108] and was found to target five upregulated proteins (CDC37, CDKN1B, GRB2, RASSF1, and SMAD2). Bosutinib had a high score of both drug suitability (dscore) and gene significance (gscore). Finally, vorinostat, romidepsin, and panobinostat were also characterized by high dscore and gscore due to their capacity to bind to the histone deacetylase inhibitors HDAC2 and HDAC7 as well as SMAD2. Another interesting candidate characterized by high values of dscore and gscore is regorafenib [111], which targeted four proteins of our dataset (CDC37, GRB2, RASSF1, and SMAD2) and represents a targeted therapy associated with limited toxicity.

Group 2 genes directly targeted an upregulated protein albeit with a slightly lower gscore, and contained the following drugs: Bortezomib, paclitaxel, venetoclax, eribulin mesylate, and docetaxel. Bortezomib is perhaps the best candidate out of this group as it a targeted therapy already used for the treatment of multiple myeloma [112] and mantle cell lymphoma [113]. Bortezomib targeted 11 proteins, including 8 proteasome subunits (BLC2, CDC37, PSMA3, PSMB1, PSMB8, PSMC2, PSMC3, PSMC4, PSMD2, PTPN2, and YWHAQ). Moreover, this group contained venetoclax, which, as mentioned earlier, is already used against high-risk del17p/mutated-TP53 CLL and patients refractory to chemotherapy [114]. Finally, paclitaxel [115], eribulin mesylate [116], and docetaxel [117] are all chemotherapeutic compounds that would likely be active against CLL cells but will associate with the typical side effects of chemotherapy.

## 4. Discussion

Technological advances over the past years have provided an extraordinary amount of data from the genome, transcriptome, and proteome analysis of cancer samples. The challenge, nowadays, is to integrate these diverse types of omics data to further comprehend complex diseases and granting more effective, safer therapeutic strategies for the application of precision medicine tailored to specific pathomolecular entities. The integration of proteomics with multi-omic data and clinical information creates a comprehensive understanding of biological processes and disease mechanisms. This integrated approach allows for the correlation of proteome abundance with transcriptome abundance and clinical outcomes, leading to more precise biomarker discovery and a better understanding of disease pathways and putative drug targets [9,118,119]. CLL is an example of complex and heterogeneous disease that lacks an effective therapy. This study combined transcriptomic, proteomic, patient survival data, and a drug repurposing approach questing for novel disease modifying factors and potential new drugs in CLL treatment and management [9].

First, we focused on the proteomic analysis of over 30 CLL patient samples and 12 healthy donors derived from 3 independent studies with publicly available data. The cumulative analysis of this data revealed that CLL cells differ from healthy cells in the regulation levels of 1023 proteins (608 upregulated and 415 downregulated). YEATS2, PIGR, BTF3, SNRPA, and NUTF2 were the top five upregulated proteins, whereas FGB, LTBP1, PPBP, GP1BA, and MPO were the most significantly downregulated proteins. Interestingly, by integrating patient survival data, we found that four out of the five top upregulated proteins (YEATS2, PIGR, SNRPA, and NUTF2) had a putative prognostic nature in CLL progression (Figure 5).

This effect is most profound for YEATS2, which is the protein with the highest upregulated value in CLL cells. YEATS2 is a subunit of the ATAC complex, which has an acetyltransferase activity on histones H3 and H4. Thus, the activity of YEATS2 offers resistance to various transcriptional repressors and enhances transcription of large gene sets, including several MAP kinases [120]. As a consequence of this function, YEATS2 overexpression has been linked with various types of cancer, including non-small cell lung cancer, head and neck squamous cell carcinoma, and pancreatic cancer [76,121,122]. Mechanistically, it has been found in previous studies that YEATS2 overexpression stimulated the PI3K/AKT pathway and altered the extracellular matrix structure, facilitating the proliferation, migration, and metastasis of hepatocellular carcinoma (HCC) cells [123]. Moreover, in non-small cell lung cancer, YEATS2 acted as a histone H3K27ac reader, orchestrating a transcriptional program critical for tumorigenesis. This program included the transcription of genes encoding ribosomal proteins, regulators of DNA replication, and components of the proteasome machinery. Additionally, we should note that WDR5, the major interactor of YEATS2, was also found to be strongly upregulated in our CLL samples. WDR5 plays a critical role in CLL by participating in the MLL/SET1 histone methyltransferase complex [124]. This complex is essential for regulating gene expression through histone methylation, particularly at histone H3 lysine 4 (H3K4), a mark associated with active transcription [125]. In CLL, dysregulation of WDR5 can lead to abnormal expression of genes involved in cell proliferation, survival, and differentiation, which are pivotal in disease progression [124]. In conclusion, further study is required to unveil the function and potential prognostic value of YEATS2 and WDR5 in CLL cells.

Similarly, another interesting candidate identified through our analysis was SNRPA. This protein belongs in the spliceosomal U1 snRNP complex, which mediates the recognition of the pre-mRNA 5′ splice-site and initiates the process of splicing in eukaryotes. Interestingly, a general defect in splicing has been recognized as a major event in CLL development through various studies in the field [39,126,127]. Thus, overexpression of SNRPA is likely a significant factor in CLL pathogenesis by contributing to splicing aberrations observed in patient cells. Similarly, PIGR was also found to be strongly upregulated in CLL cells. PIGR has been previously studied in HCC, colon cancer, pancreatic cancer, osteosarcoma, and glioma, where its high expression has been associated with unfavorable prognoses [128,129,130]. Conversely, studies have indicated favorable outcomes with PIGR expression in patients with different cancers, such as those affecting the upper gastrointestinal tract, lung, endometrium, ovaries, and breast [126,129,130]. Mechanistically, PIGR has been identified as a promoter of cellular transformation and proliferation in HCC [131]. Thus, PIGR emerges as a new factor in CLL pathogenesis that waits for further investigation. Taken together, our data propose that YEATS2, SNRPA, and, to a lesser degree, PIGR and NUTF2 may have a significant prognostic value in CLL patients.

Furthermore, to further improve the understanding of the cellular conditions existing in patient cells, we integrated transcriptomic data from 53 patients into the already analyzed proteomic datasets. The independent transcriptomic data showed very similar groups of deregulated genes, and most of them had a corresponding protein in our proteomic dataset. Nevertheless, it has been previously shown that there is limited agreement between proteomic and transcriptomic data [132]. A significant factor is the post-transcriptional regulation of protein expression, which can obscure direct correlations between mRNA levels and protein abundance [133]. Processes such as mRNA stability, translation efficiency, and post-translational modifications play crucial roles in determining the final protein composition detected in proteomic analyses [134]. Additionally, technical aspects of data acquisition and analysis contribute to the discordance. Accordingly, we observed that only a small fraction of genes had the same type of regulation when comparing the transcriptomic with the proteomic datasets (r^2^ = 0.32). This phenomenon was profound when analyzing YEATS2, which is strongly upregulated at the protein level, but merely changes in terms of abundancy at the mRNA level. As noted earlier, aberrant splicing observed in CLL cells [135] may be a key contributing factor in the identified discrepancy between mRNA and protein levels. In conclusion, this data suggests that alterations at the mRNA level should be interpreted with caution, especially if proteomic data are not available for the given cellular type.

Subsequently, we utilized our datasets to identify the major pathways involved in CLL pathogenesis by performing network and enrichment analysis of the proteomic datasets. Interestingly, both known pathways (such as RNA processing and metabolism), but also new processes (such as vesicle-mediated transport), were found to be deregulated in CLL patient cells. In total, the upregulated proteins in CLL cells clustered into groups that seem to promote stress resistance (heat-shock proteins), modify the normal metabolic routes, promote modifications of the RNA, and drive apoptosis evasion. On the contrary, the downregulated set of proteins seems to compromise the physiological immune system responses and, thus, lead to immune system evasion, as well as inflammatory signaling. The considerably overexpressed PIGR protein (Figure 5B), a recognized pro-inflammatory molecule involved in immunoglobulin trafficking across the cellular membrane [136], is likely to contribute to this trend. Conclusively, diverse proteins groups generate a distinct environment in CLL cells that influences important processes, including improved stress resistance, RNA modification in the spliceosome, apoptosis evasion, and promoting cancer progression.

Finally, as previously noted [44], drug repurposing is a feasible and cost-effective approach to identify novel therapies. Published examples in CLL include the use of antihistamines with ibrutinib [46], or the administration of single compounds, such as nelfinavir and chloroquine [137]. In the current study, we used an in silico approach, utilizing existing results originating from in vitro experiments to bioinformatically correlate deregulated proteins and pathways with chemical compounds. This analysis led to the identification of a wealth of compounds that are already used in CLL (such as venetoclax), but also new drugs with potential therapeutic use in CLL.

These drugs include the chemotherapeutic agents arsenic trioxide and romidepsin, which are characterized by high score values but are associated with the typical side-effects of chemotherapy. Moreover, we identified various targeted therapies, such as bosutinib (CML [108], vorinostat (CTCL) [138], panobinostat (multiple myeloma) [139], and regorafenib (colon, intestine, rectum, and stomach cancer [110,140]) and bortezomib (multiple myeloma and MCL) [141]. Bosutinib is a tyrosine kinase inhibitor that targets ABL and SRC family kinases, which may disrupt signaling pathways involved in cell proliferation and survival [142]. In our dataset, bosutinib targeted the following five proteins, CDC37, CDKN1B, GRB2, RASSF1, and SMAD2 (Table 1), which collectively regulate growth, proliferation, differentiation, and apoptosis [143,144,145]. Notably, there is an additional example were bosutinib was similarly repurposed against neuroblastoma, where it was shown capable of suppressing oncogenic activity via targeting multiple signaling pathways including Src/Abl and PI3K/AKT/mTOR, MAPK/ERK, and JAK/STAT3 [146]. On the other hand, Vorinostat and Panobinostat may exert a different role in CLL cells by affecting histone acetylation through targeting HDAC2, HDAC7, and SMAD2 (Table 1). By modulating these factors, Vorinostat and Panobinostat likely alter chromatin structure, impacting gene expression and cellular processes. As a matter of fact, the histone modification pathway was found significantly altered in our overrepresentation analysis (Figure 8A), thus explaining the potential mechanism of action and the activity of these drugs. Moreover, bortezomib shows potential for repurposing in CLL treatment due to its ability to target various upregulated proteasome components (Figure 8A) and modulating BCL2-mediated apoptosis. Taken together, these compounds are characterized by their targeted nature, which is associated with minimal or no toxicity. Conclusively, our analysis suggests that further exploration of vorinostat, pabinostat, regorafenib, and bortezomib in CLL might be of high research and clinical interest in experimental models of CLL.

Nevertheless, our study has certain limitations, as it relied exclusively on publicly available datasets. First, the varying sample collection methods, patient demographics, and clinical conditions across studies are factors that complicate data analysis and validation. This limitation is evident with the PDS3 proteomic dataset, which employed a different proteomic approach and thus reported fewer proteins than the PXD002004 and PXD006578 datasets. To overcome this inconsistency, we decided to only include proteins modified in the same direction (up- or down-regulation) in at least two of the three studies. There is also the possibility of selection bias, as the datasets analyzed may preferentially include certain patient subgroups, potentially skewing the results. To address this issue, we employed and analyzed data of varying techniques (including transcriptomics and patient survival) to reduce selection bias issues. Taken together, though our study has certain limitations, we believe that the use of multiple datasets of different techniques provides insights into the different manifestations and subtypes of CLL, addressing the diverse biological and clinical landscapes of the disease.

## Figures and Tables

**Table 1:** The table shows the best drugs candidates repurposed in CLL based on our bioinformatic analysis. Information about the drug name, target of the overexpressed proteins, type of drug-target interaction, their main mechanism of action, FDA drug indication and possible indication in CLL based on already existing data are provided in each column.

Drug	Target	Type of Interaction	Mechanism of Action	Indication	Possible Indication in CLL	Reference
Arsenic trioxide	CARD11, CDC37, CDKN1B, PRKCB, PTPN2, RUVBL1, SMAD2 and TRAF3	pathway inhibitor	not completely understood causes morphological changes and DNA fragmentation	promyelocytic leukemia	CT PhIII	[85]
Bosutinib	CDC37, CDKN1B, GRB2, RASSF1 and SMAD2	pathway inhibitor	inhibits the activity of the oncogenic Bcr-Abl kinase and Src-family of kinases such as Src, Lyn and Hck	CML	-	[86]
Vorinostat	HDAC2, HDAC7 and SMAD2	pathway inhibitor	inhibition of HDAC activity	CTCL	CT PhII	[87]
Romidepsin	HDAC2, HDAC7 and SMAD2	pathway inhibitor	HDAC inhibitor	CTCL	CT PhI	[88]
Panobinostat	HDAC2, HDAC7 and SMAD2	pathway inhibitor	deacetylase (DAC) inhibitor	multiple myeloma	CT PhII	[89]
Belinostat	HDAC2, HDAC7 and SMAD2	pathway inhibitor	histone deacetylase (HDAC) inhibitor	PTCL	experimental	[90]
Regorafenib	CDC37, GRB2, RASSF1 and SMAD2	pathway inhibitor	tyrosine kinase inhibitor	colorectal cancer, gastrointestinal stromal tumors, hepatocellular carcinoma	CT PhI	[91]
Palbociclib	CDKN1B and SMAD2	pathway inhibitor	inhibition of cyclin D-CDK4/6 complex activity	breast cancer	CT PhI	[92]
Imatinib	GRB2 and SMAD2	pathway inhibitor	inhibits the activity of the oncogenic Bcr-Abl kinase and other kinases	leukemias, myelodysplastic/myeloproliferative disease, systemic mastocytosis, hypereosinophilic syndrome, dermatofibrosarcoma protuberans, gastrointestinal stromal tumors	CT PhII	[93]
Ponatinib	GRB2 and SMAD2	pathway inhibitor	multi-target kinase inhibitor	CML	CT PhI	[94]
Ribociclib	CDKN1B and SMAD2	pathway inhibitor	inhibitor of cyclin-dependent kinase (CDK) 4 and 6	breast cancer	-	[95]
Abemaciclib	CDKN1B and SMAD2	pathway inhibitor	inhibits CDK4 and CDK6	breast cancer	CT PhI	[96]
Nilotinib	SMAD2	pathway inhibitor	inhibits the activity of the oncogenic Bcr-Abl kinase	CML	-	[97]
Dasatinib	SMAD2	pathway inhibitor	inhibition of BCR-ABL, SRC family (SRC, LCK, YES, FYN), c-KIT, EPHA2, and PDGFRβ	CML, ALL	CT PhII	[98]
Axitinib	SMAD2	pathway inhibitor	VEGFR and kinase inhibitor	renal cell carcinoma	-	[99]
Valproic acid	HDAC2, HDAC7 and SMAD2	pathway inhibitor	inhibitor of GABA, impacts the extracellular signal-related kinase pathway (ERK), impact fatty acid metabolism, HDAC inhibitor	anticonvulsant	CT PhII	[100]
Phenylbutyric acid	HDAC2, HDAC7 and SMAD2	pathway inhibitor	conjugated with phenylacetyl-CoA	urea cycle disorders	experimental	[101]
Bortezomib	BCL2, CDC37, PSMA3, PSMB1, PSMB8, PSMC2, PSMC3, PSMC4, PSMD2, PTPN2 and YWHAQ	direct inhibitor	inhibitor of the 26S proteasome	multiple myeloma, MCL	CT PhI	[102]
Paclitaxel	BCL2, CDC37, MAP2, MAP4, PTPN2 and YWHAQ	direct inhibitor	interferes with the normal function of microtubule growth	Kaposi’s sarcoma and cancer of the lung, ovarian, and breast	CT PhII	[103]
Venetoclax	BCL2, CDC37, PTPN2 and YWHAQ	direct inhibitor	BCL-2 inhibitor	CLL, SLL, AML	FDA aproved	[104]
Eribulin Mesylate	BCL2, CDC37, PTPN2 and YWHAQ	direct inhibitor	microtubule inhibitor	breast cancer	-	[105]
Docetaxel	BCL2, CDC37, MAP2, MAP4, PTPN2 and YWHAQ	direct inhibitor	interferes with the normal function of microtubule growth	breast cancer, NSCL, prostate cancer, gastric adenocarcinoma, head and neck cancer	CT PhI	[106]

## Data Availability

All data are available at Supplementary Materials.

## Supplementary Materials

The following supporting information can be downloaded at: https://www.mdpi.com/article/10.3390/jpm14080831/s1, Supplementary figures pdf file; Supplemental Table S1: (Selected-omics datasets).xlsx; Supplemental Table S2: (Processed data).xlsx; Supplemental Table S3: (Merged data).xlsx; Supplemental Table S4: (Proposed drugs).xlsx.

## Abbreviations

BioDBnet	the biological database network
CML	Chronic myeloid leukemia
CLL	Chronic Lymphocytic Leukemia
CT PhI	Clinical Trial Phase I
CT PhII	Clinical Trial Phase II
CT PhIII	Clinical Trial Phase III
CTCL	Cutaneous T-cell lymphoma
FDA	Food and Drug Administration
GEO	Gene Expression Omnibus
GTEx	Genotype-Tissue Expression
GO	Gene Ontology
ICGC	International Cancer Genome Consortium
IGHV	immunoglobulin heavy chain variable region gene
INDELs	Insertion-deletion mutations
KEGG	Kyoto Encyclopedia of Genes and Genomes
Log2(FC)	Log2[(measurement of proteins/genes of CLL patients)/(measurement of proteins/genes of healthy donors)]
M-CLL	mutated CLL
MCL	mantle cell lymphoma
PCA	Principal Component Analysis
PIGR	Polymeric immunoglobulin receptor
PRIDE	PRoteomics IDEntifications database
SNPs	Single nucleotide polymorphisms
SNRPA	Small nuclear ribonucleoprotein polypeptide A
STRING	Search Tool for the Retrieval of Interacting Genes/Proteins
SWATH	Sequential Window Acquisition of all Theoretical Spectra
TCGA	Cancer Genome Atlas
U-CLL	unmutated CLL
YEATS2	YEATS domain-containing protein 2
