# Supplementary material for: Integrative Analysis of Multi-Omics Data to Identify Deregulated Molecular Pathways and Druggable Targets in Chronic Lymphocytic Leukemia
Source: J Pers Med. 2024 Aug 6;14(8):831. doi: 10.3390/jpm14080831 (PMC11355716; doi:10.3390/jpm14080831)
Supplement: Supplementary file 1 [file jpm-14-00831-s001.zip › Supplementary figures.pdf]

Supplementary Figures

Integrative analysis of multi-omics data to identify deregulated molecular pathways and druggable targets in Chronic Lymphocytic Leukemia

Dimitra Mavridou 1,2,3, Konstantina Psatha 2,3,4, Michalis Aivaliotis 1,2,3

1. Laboratory of Biochemistry, School of Medicine, Faculty of Health Sciences, Aristotle University of Thessaloniki, GR-54124 Thessaloniki, Greece
2. Functional Proteomics and Systems Biology (FunPATh)—Center for Interdisciplinary Research and Innovation (CIRI-AUTH), GR-57001 Thessaloniki, Greece
3. Basic and Translational Research Unit, Special Unit for Biomedical Research and Education, School of Medicine, Aristotle University of Thessaloniki, GR-54124 Thessaloniki, Greece
4. Laboratory of Medical Biology – Medical Genetics, School of Medicine, Faculty of Health Sciences, Aristotle University of Thessaloniki, GR-54124 Thessaloniki, Greece

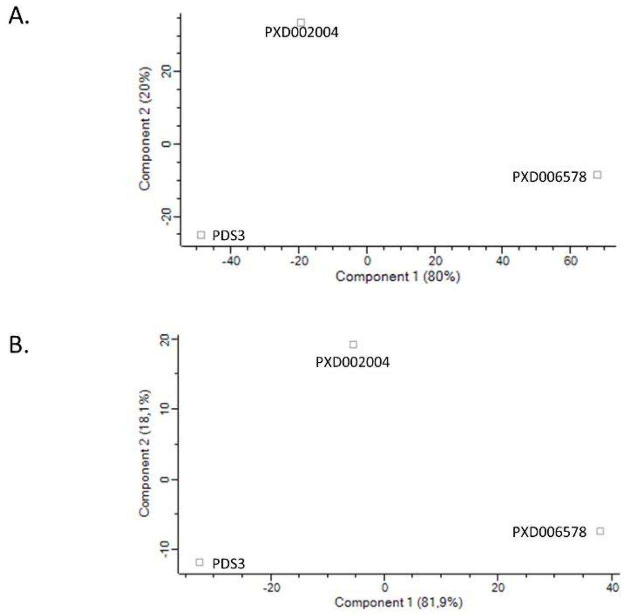

Supplemental Figure S1: PCA of the three proteomic datasets (A) in all the proteins identified and (B) in the common differentially expressed proteins. It is observed that no one dataset is particularly related to another one both in the total proteins identified and in the common differentially expressed proteins, each dataset has a distinct profile showing the importance of each dataset in the holistic unbiased approach to proteomics. As it makes sense in common differentially expressed proteins the datasets become more relevant.

Supplementary Figures

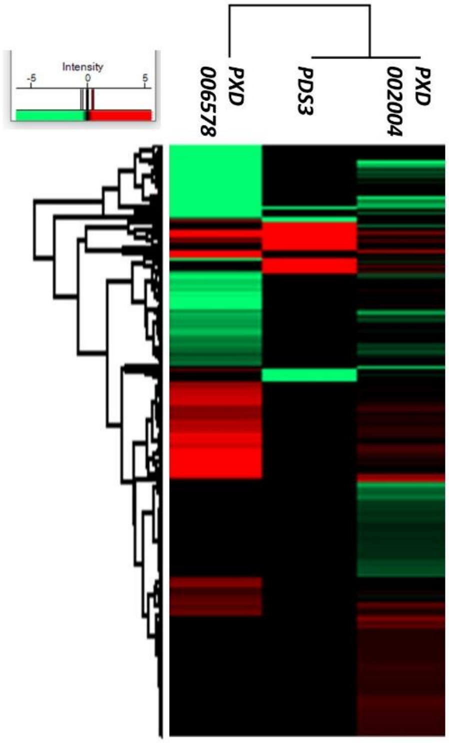

Supplemental Figure S2: Heatmap of the total proteins identified in the three datasets. Not all proteins were detected in all datasets and there are some proteins that do not be expressed in the same way in all studies.

Supplementary Figures

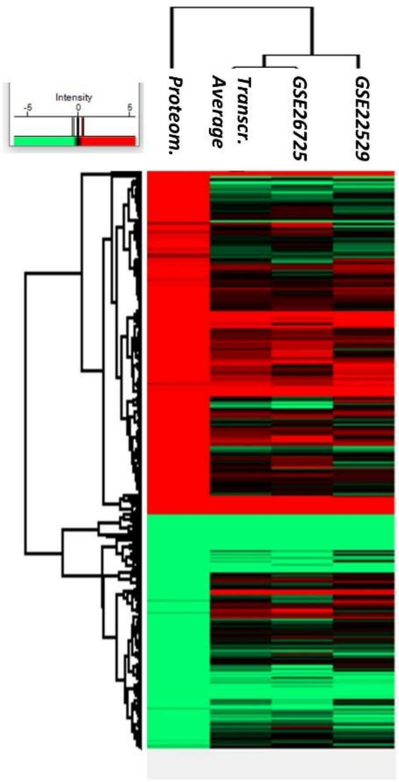

Supplemental Figure S3: Heatmap of the common identifiers between the transcriptomic datasets and the means of common differentially expressed proteins. Another diagram that visualizes once again the different regulation of genetic information between proteomics and transcriptomics.
